# SARS-CoV-2 infection and complicated appendicitis in adults in Lima, Peru: a matched case-control study

**DOI:** 10.1186/s12893-025-02897-7

**Published:** 2025-04-16

**Authors:** Agustín Mansilla-Sandoval, Diana Corrales-Delgado, Zully M. Puyén, Percy Mansilla-Doria, Edwin Orendo-Velásquez, Luis Huicho, Diego Fano-Sizgorich

**Affiliations:** 1 Facultad de Ciencias de la Salud, Universidad Peruana de Ciencias Aplicadas, Lima, Peru; 2 Servicio de Cirugía General, Hospital de Emergencias Grau– EsSalud, Lima, Peru; 3 Centro de Investigación en Salud Materna e Infantil, Centro de Investigación para el Desarrollo Integral y Sostenible and Facultad de Medicina, Universidad Peruana Cayetano Heredia, Lima, Peru; 4 Laboratorio de Endocrinología y Reproducción, Laboratorios de Investigación y Desarrollo, Facultad de Ciencias e Ingeniería, Universidad Peruana Cayetano Heredia, Lima, Peru

**Keywords:** Complicated appendicitis, Acute appendicitis, SARS-CoV-2, COVID-19, Latin America

## Abstract

**Background:**

Acute appendicitis may be uncomplicated or may present with life threatening complications. Since the outbreak of the COVID-19 pandemic, there has been an increase in the number of cases of complicated appendicitis, suggesting a possible association between them. Therefore, we aimed to determine the association between SARS-CoV-2 infection and complicated appendicitis in surgical patients in Lima, Peru, from March 2020 to December 2021.

**Methods:**

A matched case-control study was conducted. Clinical records of patients ≥ 18 years old who underwent surgery for appendicitis and had at least one positive SARS-CoV-2 diagnostic test were selected. Patients undergoing surgery for complicated appendicitis were considered cases, and patients undergoing surgery for uncomplicated appendicitis were controls. A 1:1 matching by sex, age, and month of surgery was performed. Conditional logistic regression modeling was performed to calculate crude and adjusted conditional odds ratios (cOR).

**Results:**

The positivity rate for COVID-19 tests was 73.6% for cases and 26.4% for controls. The crude cOR was 4.88 (95% IC 2.89–8.23, *p* < 0.001), and the adjusted cOR was 3.52 (95%IC 1.82–6.81, *p* = 0.001), after controlling for onset time of symptoms and awaiting time before surgery.

**Conclusions:**

Surgery for complicated appendicitis was associated with SARS-CoV-2 infection. Patients with this infection may be at higher risk of complicated appendicitis and thus may need additional clinical monitoring.

**Supplementary Information:**

The online version contains supplementary material available at 10.1186/s12893-025-02897-7.

## Background

Acute appendicitis is one of the most frequent causes of acute abdominal pain needing emergency surgery [1]. It occurs frequently between the second and third decades of life, with a frequency ranging from 7 to 8%, although with geographical variations [1]. The most frequent symptoms are pain in the right iliac fossa, nausea and/or vomiting, loss of appetite, fever, and pain on rebound or decompression on physical examination [2].

Acute appendicitis is considered an inflammatory process that can be triggered by obstruction of the cecal appendiceal lumen caused by hypersecretion, distension of the appendix, bacterial overgrowth or infectious agents [3, 4]. The diagnosis is typically clinical, with complementary laboratory and imaging tests [5]. Different factors have been associated with appendicitis, including sociodemographic variables such as gender [6], age, marital status and education; and life-style factors such as daily meat intake, low fiber-rich vegetable consumption and smoking [7].

Complicated appendicitis can be often difficult to manage, it has increased morbidity and 30–50% of surgical wound infection [8]. Factors that have been associated with complicated appendicitis include immunosuppression, severe malnutrition, uncompensated diabetes and extreme ages of life [9]. Biomarkers such as high leukocytes count and C-reactive protein have been associated with gangrenous appendicitis [10]. Another important associated factor is the delay in the diagnosis [7], or a longer interval between the onset of symptoms and admission [11].

COVID-19 disease is produced by the SARS-CoV-2 virus, identified in late 2019. This virus produces molecular changes and affects mainly the lungs, but it can also affect other organs [12]. SARS-CoV-2 infects cells by binding to the ACE2 receptors [12], which are found in several cell groups including the large bowel enterocytes [13].

There are a few studies, all ecological, reporting an increase in the number of cases of complicated appendicitis during the pandemic [14, 15], which suggest that SARS-CoV-2 infection could be acting as a risk factor. In a series of case-reports it was found that SARS-CoV-2 may dysregulate interferon production and cytokine activation, disrupting immune tolerance and possibly leading to a higher risk of related diseases such as inflammatory bowel disease [16]. On the other hand, there are reports documenting delayed health care during the Covid-19 pandemic in different countries [17], including Peru [18]. Such delay could have resulted in conditions like complicated appendicitis.

We aimed in this study to evaluate the association between SARS-CoV-2 infection and complicated appendicitis among patients attending the emergency ward of a Peruvian hospital from March 2020 to December 2021.

## Materials and methods

### Study setting

In brief, the Peruvian health system in charge of providing health services comprises the Comprehensive Health Insurance (Seguro Integral de Salud, SIS) run by the Ministry of Health that covers the uninsured segment of the population, the social security (EsSalud) that covers the employees, the Sanidad de las Fuerzas Armadas y Policiales covering the military and the police members, and the private sector.

### Study design and study population

Hospital de Emergencias Grau is a teaching hospital that belongs to EsSalud and is located in Lima, the capital city of Peru. It attends an average of 600 people per day distributed in all specialties, including Surgery and Trauma. We conducted a matched case-control study of patients with acute appendicitis. Clinical records of the patients were accessed through the surgery department.

Patients were included if they were 18 years old or more and had at least one COVID-19 test registered in their clinical records by the time of surgery. The exclusion criteria were pregnant women and incomplete clinical records. An incomplete clinical record was considered as that lacking information on age, sex, acute appendicitis diagnosis, surgery date, and COVID-19 test. Overall, there were 504 eligible clinical records for the case-control matching.

### Definition of uncomplicated and complicated appendicitis

Appendicitis is classified into complicated and uncomplicated, depending on the severity of the appendix inflammation and the possible complications that may arise [19]. In this study, uncomplicated appendicitis was defined as a condition in which the appendix is inflamed but has not perforated or caused a more extensive infection (ICD-10 code K35.9). Complicated appendicitis was defined as a condition in which the appendix becomes necrotic or perforated, that led to localized or generalized infection in the abdomen (peritonitis) or to abscess formation (ICD-10 code K35.1. K35.3 or K35.0). Complicated appendicitis requires more urgent surgical intervention and poses a higher risk of postoperative complications, prolonging recovery time and in some cases requiring additional treatment such as abscess drainage.

### Definition of SARS-CoV-2 infection

During the pandemic, whenever a patient with abdominal pain arrived to the emergency ward, he/she was fully assessed, including the request of a COVID-19 diagnostic test.

A SARS-CoV-2 infected participant was defined as a patient with a positive quantitative-real-time polymerase chain reaction method (q-RT-PCR) or a positive COVID-19 antigenic test. A chest x-ray or a thorax CT scan was requested when a Covid-19 test was not available and the patient had respiratory symptoms, oxygen saturation below 90%, fever in the last 2 weeks, or had been in contact with a covid patient.

The COVID-19 antigenic test was performed by swabbing the patient’s nostrils, mixing the sample with the kit buffer, and finally placing the solution in a test cassette. A positive result was considered when two red brands (control and antigen) appeared. A positive q-RT-PCR result was determined according to the Pan American Health Organization recommendations [20].

The chest x-ray and thorax CT scan were considered abnormal when pulmonary consolidation or ground-glass opacity were found. Since these tests do not confirm the SARS-CoV-2 infection status, a nasopharyngeal swabbing sample was obtained for further confirmation through q-RT-PCR. The SARS-CoV-2 infection status of these patients was defined on the basis of the q-RT-PCR confirmatory test.

Information on COVID-19 diagnostic tests used for each patient was present in the clinical record.

### Clinical information

Available clinical information included symptoms onset time (SOT) and awaiting time before surgery (ATBS). SOT was defined as the time interval from the beginning of symptoms until hospital admission (hours), while ATBS was defined as the time interval since admission until surgery.

### Laboratory panel

Data on whole blood cell counts, blood biochemistry, serum glucose, creatinine and procalcitonin was retrieved from the clinical records.

### Causal pathway

**Fig. 1:**
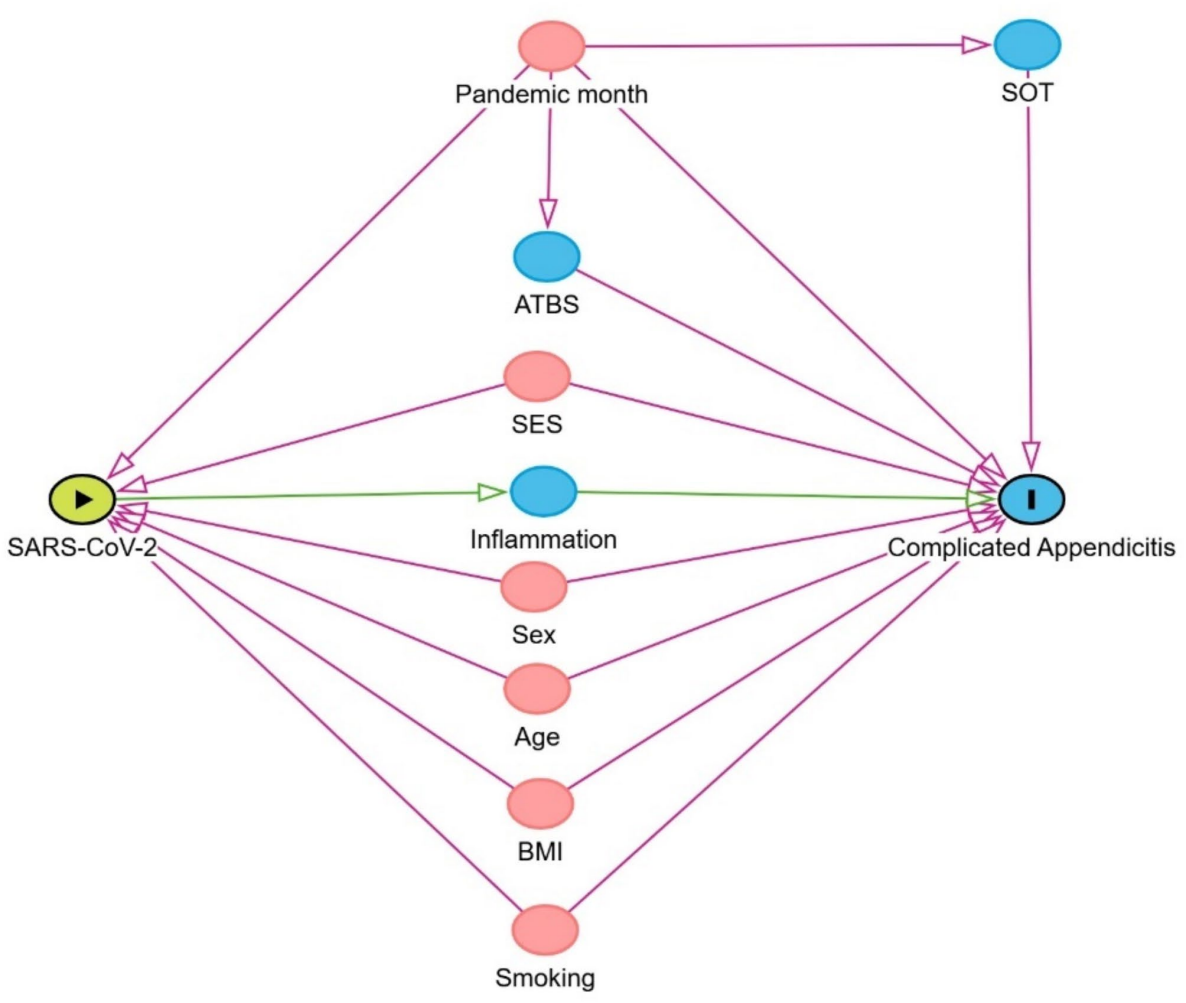
Proposed causal pathway. The green circle with an arrow inside is the exposure variable. The blue circle with an “I” inside is the outcome variable. Blue circles are observed variables. Red circles are confounders. A direct arrow from one variable to another denotes a causal relationship. SOT: symptoms onset time. ATBS: awaiting time before surgery. SES: socio-economic status. BMI: body mass index

As a guide to our analyses, we built a direct acyclic graph (DAG) causal pathway for complicated appendicitis, based on the published literature [7, 9, 11] (Fig. 1). The DAG was constructed using the DAGitty software [21].

In this proposed pathway, pandemic month, socio-economic status, smoking, body mass index, sex and age are considered confounders. Pandemic month was considered as a factor having a direct causal association with symptoms onset time, and with awaiting time before surgery. Socio-economic status, smoking habits and body mass index data were not available. Inflammation-related and metabolic biomarkers are considered mediating variables in the causal pathway.

### Cases and controls definition and matching

A case was defined as a patient who underwent surgery for complicated appendicitis, while a control was defined as a patient who underwent surgery for uncomplicated appendicitis. A 1:1 case-control matching was performed, with sex, age quartile, and month of surgery included as matching variables. A total of 201 matched pairs were considered for the analysis and 102 eligible participants were excluded due to matching incompatibility.

### Statistical analysis

Median and interquartile ranges were obtained for numeric variables. For categorical variables, absolute and relative frequencies were calculated. Bivariate analysis of patients’ characteristics was performed to evaluate the differences between cases and controls. For numerical variables, the paired Student’s t-test or the Wilcoxon’s sign-rank test was used, while for the SARS-CoV-2 infection status the McNemar test was used.

Crude (ccOR) and adjusted (acOR) conditional logistic regression models were run to assess the association between SARS-CoV-2 infection and complicated appendicitis. In the adjusted model, additional to the matching variables, the regressions were controlled for symptoms onset time and awaiting time before surgery. Biomarkers were not considered as control variables since they are part of the causal pathway (mediators) between SARS-CoV-2 infection and complicated appendicitis. We additionally stratified the conditional regression analysis in COVID-19 pre- (March 2020– February 2021) and post-vaccine (March 2021– December 2021) periods. A *p*-value < 0.05 was considered as statistically significant.

All analyses were performed using the STATA statistical software (v. 18.0).

## Results

Overall, 140 patients (34.8%) had a positive COVID-19 test by the time of surgery. Most participants were screened with an antigen COVID-19 test (47.5%) or a molecular q-RT-PCR test (31.8%). Median age was 39 years, and 228 subjects were males. Median of symptoms onset time and awaiting time before surgery were 24 and 3 h, respectively. Median serum glucose before surgery was 112 mg/dL, and mean procalcitonin was 0.23 ng/mL (Table 1).

**Table 1:** Paired study sample characteristics

Variable		Median (IQR)
SARS-CoV-2 infection*		
Yes		140 (34.8)
No		262 (65.2)
Age (years)		39 (21)
Sex*		
Female		174 (43.3)
Male		228 (56.7)
Symptoms onset time (hours)		24 (24)
Awating time before surgery (hours)		3 (3)
Glucose (mg/dL)		112 (27)
Urea (mg/dL)		25 (12)
Creatinine (mg/dL)		0.89 (0.3)
Procalcitonin (ng/mL)		0.23 ± 0.1
White blood cells (x10^9^/L)		13.31 ± 4.7
Segmented neutrophils (%)		83 (11)
Lymphocytes (%)		11 (11)
COVID-19 test*		
Antigen		191 (47.5)
Molecular (q-RT-PCR)		128 (31.8)
Chest x-ray**		16 (4.0)
Thorax CT scan**		67 (16.7)

IQR: Interquartile range. q-RT-PCR: Quantitative real-time polymerase chain reaction.

*Number (%)

** Participants with an abnormal chest x-ray or thorax CT scan had a confirmatory q-RT-PCR test

The results of the bivariate analyses are shown in Table 2. There was a strong association between SARS-CoV-2 infection and complicated appendicitis (*p* < 0.001), with 73.6% SARS-CoV-2 positivity in cases and 26.4% in controls. The symptoms onset time was 48 h for cases and 24 h for controls (*p* < 0.001). Biomarker levels such as glucose, white blood cell count, and proportion of segmented neutrophils were higher in cases (*p* < 0.05), while proportion of lymphocytes was higher in controls (*p* < 0.001).

**Table 2:** Bivariate associations in matched cases and controls

Independent variable		Cases	Controls	*p*-value
		Median (IQR)	Median (IQR)	
SARS-CoV-2 infection*				< 0.001
Yes		103 (73.6)	37 (26.4)	
No		98 (37.4)	164 (62.6)	
Symptoms onset time (hours)		48 (16)	24 (6)	< 0.001
Awaiting time before surgery (hours)		3 (3)	3 (3)	0.875
Glucose (mg/dL)		115 (32)	109 (23)	0.002
Urea (mg/dL)		26 (11)	25 (12)	0.197
Creatinine (mg/dL)		0.89 (0.3)	0.9 (0.3)	0.681
Procalcitonin (ng/mL)		0.2 (0.1)	0.2 (0.1)	0.119
White blood cells (x10^9^/L)**		14.5 ± 4.6	12.8 ± 4.6	< 0.001
Segmented neutrophils (%)		83 (9)	81 (13)	< 0.001
Lymphocytes (%)		10 (8)	13 (13)	< 0.001

IQR: Interquartile range

*n(%). Assessed through McNemar test

**Mean ± standard deviation. Assessed through paired Student’s t-test

Median (IQR) variables were assessed through Wilcoxon’s sign-rank test

Results of crude and adjusted logistic regressions are shown in Table 3. SARS-CoV-2 infection was significantly associated with complicated appendicitis in both the crude (*p* < 0.001) and adjusted (*p* = 0.001) models in the whole study period, respectively, with cases having 3.52 (95%CI 1.82–6.8) higher risk of being infected with SARS-CoV-2 compared to controls in the adjusted logistic regression analysis. When stratifying the analysis by pre- and post-vaccine periods, there was no significant association between SARS-CoV-2 infection and complicated appendicitis in the pre-vaccine period (*p* = 0.557), while in the post-vaccine period the association was significant (acOR = 7.18, 95%CI 1.88–27.45, *p* = 0.004). Symptoms onset time and awaiting time before surgery were significant risk factors in the adjusted regressions, both in the pre-vaccine and post-vaccine periods.

**Table 3:** Crude and adjusted conditional regression

Variable	Whole study period				Pre-vaccine period	Post-vaccine period
	ccOR (95%CI)			acOR (95%CI)	ccOR (95%CI)	acOR (95%CI)
SARS-CoV-2 infection	4.88 (2.89–8.23)*			3.52 (1.82–6.81)*	0.57 (0.08–4.05)	7.18 (1.88–27.45)*
Symptoms onset time	1.09 (1.06–1.11)*			1.08 (1.06–1.11)*	1.13 (1.02–1.25)*	1.10 (1.05–1.15)*
Awaiting time before surgery	1.02 (0.98–1.07)			1.04 (0.96–1.12)	1.12 (0.88–1.41)	1.00 (0.85–1.18)

Pre-vaccine period: March 2020- February 2021

Post-vaccine period: March 2021-December 2021

ccOR: Crude conditional odds ratio

acOR: Adjusted conditional odds ratio

*p-value < 0.05

## Discussion

We found in our study that SARS-CoV-2 infection is strongly associated with complicated appendicitis, increasing the odds by more than 3 times, compared to non-infected patients. Symptoms onset time and awaiting time before surgery remained significant risk factors, suggesting that COVID-19 may exert its effect on the outcomes, at least in part, by delaying the diagnosis and the time of surgical management of patients.

However our results are in contrast with those from another study in Peru, which did not find an association between COVID-19 positivity with appendix perforation or necrosis, and even found lower odds for peritonitis, abscess and plastron in the COVID-19 positive group [22]. This difference might be explained by the matching criteria used. We matched cases and controls by sex, age, and month of surgery, whereas the other study matched cases and controls using only the week of the year in which surgery was performed. Matching by month of surgery, sex, and age helps make cases and controls more similar in terms of demographics and seasonal patterns. However, matching by week of surgery might better account for how the pandemic affected the hospital’s ability to perform timely emergency surgeries [23]. On the other hand, the study performed in Cusco (3600 m above sea level) suggested that the lower atmospheric pressure found at high altitude might be associated with a lower risk of acute appendicitis [24, 25]. Also, previous studies have suggested that high-altitude might confer a lower risk for COVID-19 death [26], although the evidence is not conclusive [27]. It is far from clear how altitude of residence or other environmental factors may modify the risk of appendicitis and complications, thus further investigation on this topic is warranted.

Infection with SARS-CoV-2 might increase the risk of acute appendicitis, as found in a population-based cohort study in Sweden, in which the overall risk of acute appendicitis within 21-days after a SARS-CoV-2 infection was 1.68 [28]. Additionally, different studies have reported an increase in the frequency of complicated appendicitis during the pandemic. For instance, in a report from China the prevalence of complicated appendicitis was 51.7% vs. 12.4% in the pre-pandemic period [29]. A similar trend was observed in Massachusetts, USA, where the prevalence of perforated and gangrenous appendicitis increased by 21% and 29%, respectively [14]. In Israel an increase in the presentation of perforated appendicitis was observed [30]. A meta-analysis that included 3559 patients found a relative risk of 1.55 for complicated appendicitis during the pandemic [31]. Additionally, the severity of appendicitis symptoms was found to be higher during the pandemic [32].

It is hypothesized that the increase in the incidence and prevalence of complicated appendicitis and of the severity of symptoms is due to the longer time of symptoms onset during the pandemic [33], which were found to be more than 10 h higher [34] and even 48 h longer when compared to the period prior to the pandemic [33]. We found in our study that the cases had longer symptoms onset time, nonetheless in the multivariate regression analysis, after controlling symptoms onset time and awaiting time before surgery, the higher odds for complicated appendicitis was maintained.

On the other hand, the higher risk of complicated appendicitis reported during the pandemic may be due to the fact that the health facilities were not able to provide appropriate and timely healthcare due to overflow of patients, lack of resources, or restricted access, or to reluctance of patients to seek health care because of fear of contagion [35, 36]. This potential confounding was overcome in our study by matching the cases and controls by the month the surgery took place, allowing us to control for the indirect effects of the pandemic on the timely provision of health services. It remains to be determined whether this association has changed and whether the rates of appendicitis and complicated appendicitis have shifted in the post-COVID-19 era.

Of note, the OR was higher for complicated appendicitis in the post-vaccine period. Acute abdomen cases have been reported in the post-vaccine period [37], although vaccine-related acute appendicitis characteristics are not different from the non-vaccine-related cases [38]. Nationwide analyses found no association between COVID-19 mRNA vaccination and acute appendicitis [39, 40]. Inactivated virus, mARN and recombinant viral vector COVID-19 vaccines were administered in Peru during the study period, beginning on February 2021 [41], nonetheless, the patients’ vaccination status was not evaluated due to the lack of such information. This should be taken into account in future studies.

SARS-CoV-2 infects cells by binding to the ACE2 receptor, which is widely distributed in different tissues and organs, including epithelial cells of the large bowel [42]. It is possible that a previous or current infection may produce an inflammatory response in this organ [16], which could increase the severity of appendicitis cases [43, 44]. On the other hand, there were different SARS-CoV-variants identified in Peru during the pandemic, which showed a different risk of clinical outcomes such as death [45], being Lambda variant the one that predominated during the first half of 2021 [45, 46], and Delta during the second half of 2021 [45]. It is possible that the different SARS-CoV-2 variants might have a different risk of acute or complicated appendicitis, and this should be considered in future studies.

The role of SARS-CoV-2 in patient outcomes extends beyond surgical outcomes to long-term consequences such as post-surgical complications and hospital readmissions. Increased length of hospital stay and higher rates of reoperation after appendectomy have been reported during the pandemic [47], although findings across studies remain inconsistent [48, 49].

Although our study provides evidence on the association between SARS-CoV-2 and complicated appendicitis during the pandemic period even after controlling for an important covariate such as symptoms onset time, we acknowledge some limitations. Firstly, our patients came from one single hospital, therefore generalizability to other health facilities of different complexity may be limited. In a study done in another district of Lima, the prevalence of complicated appendicitis in SARS-CoV-2 infected patients was 67% [50], which is similar to our results, possibly meaning that unaddressed factors such as differences in healthcare access of the study population might be comparable with those in other parts of Lima; nonetheless, future studies should try to include data from different centers. Secondly, there is a potential selection bias since the excluded patients might be different from those included. However, when we compared age, sex, symptoms onset time and awaiting time before surgery between included and excluded patients, they were not different (Supplementary material 1). Additionally, it is possible that some people with uncomplicated appendicitis might have avoided surgery due to fear of contagion, especially considering that there was a higher prevalence of moderate depressive symptoms in Peru during and after the pandemic [51]. Thirdly, data on comorbidities such as diabetes mellitus type 2, obesity, lifestyle such as nutrition and smoking were not available. Also, data on the socio-economic status of patients and on their district of residence was not available, although we may assume a quite homogeneous socioeconomic status of the study population, as all patients were formal employees. Lack of those important confounders and mediators may limit the accuracy of our results. We had no information about COVID-19 symptoms and therefore could not assess if COVID-19 severity or previous infections with this agent might have contributed to the appendicitis severity. The fact that our study was conducted at a single hospital may limit the generalizability of the results to other regions or countries, as other factors such as differences in healthcare access, the burden of COVID-19 and medical infrastructure may have affected the outcomes. Although we did not address these factors in our study, our temporal matching criteria may have reduced at least in part the differences in the burden of COVID-19. Finally, we acknowledge that a longer follow-up period could have provided a broader understanding of the pandemic’s impact on appendicitis management.

Further research could include investigating how COVID-19 impacts hospital workflows, delays in diagnosis, and medical treatment, which may indirectly contribute to increased appendicitis complications.

The findings of this study not only provide new insights into health outcomes related to SARS-CoV-2 infection but also emphasize the need for heightened clinical awareness and increased health system resilience in the post-pandemic era. Clinicians should exert particular interest in assessing acute abdominal pain in patients with Covid-19 [52], to facilitate early diagnosis and prompt treatment of potential complications. Health facilities should optimize resource allocation during surges in COVID-19 cases, to ensure that adequate equipment and trained human resources are in place to offer timely and adequate care and to prevent conditions like complicated appendicitis.

## Conclusion

SARS-CoV-2 infection was strongly associated with complicated appendicitis, increasing more than three times the odds of this condition. Patients with this infection may be at higher risk of complicated appendicitis and thus may need additional clinical monitoring. It remains to be seen whether the implementation of public health strategies such as SARS-CoV-2 vaccination, or if the different SARS-CoV-2 variants have modified this association.

## Electronic supplementary material

Below is the link to the electronic supplementary material.

Supplementary Material 1

## Data Availability

The datasets used and/or analysed during the current study are available from the corresponding author on reasonable request.

## Abbreviations

SIS: Seguro Integral de Salud
EsSalud: Social security
SOT: Symptoms onset time
ATBS: Awaiting time before surgery
DAG: Direct acyclic graph
SES: Socio-economic status
BMI: Body mass index
ccOR: Crude conditional odds ratio
acOR: Adjusted conditional odds ratio
