# Supplementary material for: SARS-CoV-2 infection and complicated appendicitis in adults in Lima, Peru: a matched case-control study
Source: BMC Surg. 2025 Apr 16;25:159. doi: 10.1186/s12893-025-02897-7 (PMC12001637; doi:10.1186/s12893-025-02897-7)
Supplement: Supplementary file 1 — Supplementary Material 1 [file 12893_2025_2897_MOESM1_ESM.docx]

| **Supplementary material 1.** Characteristics comparison between the excluded and included participants. | | | | | |
| --- | --- | --- | --- | --- | --- |
| **Variable** | | | **Excluded** | **Included** | **p-value** |
|  |  |  | **n=367** | **N=504** |  |
|  |  | |  |  |  |
| Age | | | 47.20 ± 2.75 | 42.82 ± 0.70 | 0.061^α^ |
|  | | |  |  |  |
| Sex* | | |  |  | 0.078^λ^ |
|  | | Male | 32 (72.73) | 296 (58.76) |  |
|  | | Female | 12 (27.27) | 208 (41.24) |  |
|  | | |  |  |  |
| Symptoms onset time^β^ | | | 36 (24) | 27 (24) | 0.060^¥^ |
|  | | |  |  |  |
| Awating time before surgery^β^ | | | 3 (1.5) | 3 (3) | 0.903^¥^ |
|  |  | |  |  |  |
| *Data is presented as number of observations (n) and percentage (%). Excluded sex categories does not sum 367 participants due to missing data.  ^λ^Fisher’s exact test.  ^β^Data is presented as Median (interquartile range).  ^α^Student’s t-test.  ^¥^ Wilcoxon’s rank-sum test. | | | | | |
|  | | | | | |
